# Manufacture of Hemispherical Shell and Surrounding Eave-Shaped Electrodes

**DOI:** 10.3390/mi12070815

**Published:** 2021-07-12

**Authors:** Renxin Wang, Bing Bai, Wendong Zhang, Huiliang Cao, Jun Liu

**Affiliations:** State Key Laboratory of Dynamic Testing Technology, North University of China, Taiyuan 030051, China; wangrenxin@nuc.edu.cn (R.W.); 15135173839@163.com (B.B.); wdzhang@nuc.edu.cn (W.Z.)

**Keywords:** hemispheric shell, eave-shaped electrodes, blowing, nondestructive estimation, asymmetry, surface roughness

## Abstract

A hemispherical resonator consists of a hemispherical shell and the surrounding circular electrodes. The asymmetry of a hemispherical shell has influence on the vibrating mode and quality factor. The gap distance from shell to electrode is critical for the capacitance and sensitivity of a hemispherical resonator. To realize a symmetric shell and a small gap, a kind of micro-hemispherical resonator (μHR) structure including sandwich-shaped stacks and eave-shaped electrodes has been developed using a glassblowing process. The blowing process could bring favorable surface roughness and symmetry. The locations of the hemispherical shell and surrounding electrodes can be precisely controlled by the designs of sandwich-shaped stacks and eave-shaped electrodes, making it feasible to realize uniform and small gaps. In addition, electrical insulation between the hemispherical shell and eave-shaped electrodes can be guaranteed owing to eave-shaped structure. The fabrication process and results are demonstrated in detail. Furthermore, an estimation method of shell thickness in a nondestructive manner is proposed, with deviation below 5%. Taking asymmetry, surface roughness, and gap into consideration, these results preliminarily indicate this structure with a hemispherical shell and surrounding eave-shaped electrodes is promising in hemispherical resonator applications.

## 1. Introduction

A hemispherical resonator is a kind of axisymmetric shell resonator with advantages of high reliability, long life, and stable physical properties [1]. However, the presently used hemispherical resonator has a relatively large size and high manufacturing cost. In recent years, micro-hemispherical resonators (μHRs) have been developed. The μHR consists of a 3D micro-hemispherical shell structure and circular electrodes around the hemispherical shell [2]. Geometric imperfection characterization of the shell and electrodes is crucial for the quality of resonators [3]. Therefore, there are two difficult issues: how to fabricate a uniform hemispherical shell, and how to fulfill the alignment of the shell and surrounding circular electrodes. As for the first issue, with the development of 3D MEMS techniques, the fabrication of a 3D micro-hemispherical shell structure has become feasible, for example, via chemical vapor deposition (CVD) on the hemispherical cavity [4] or a blowing process [5,6]. The asymmetry of the shell influences the wineglass mode [7], frequency mismatch [8], and quality factor [2]. Therefore, a uniform and symmetric hemispherical shell is required.

For the second issue, the essence of alignment is the realization of assembly with a uniform and small gap. When the μHR is in working mode, it is excitated by electrostatic force, and the change of capacitance is detected to distinguish resonant frequency. Therefore, the gap between shell and electrode is critical for the intensity of driving and detection of signal. 

There are two dominant approaches to fabricate μHR. One is via chemical vapor deposition (CVD) on the hemispherical cavity, the other is via a blowing process. As for the CVD approach, a 3D micro-hemispherical shell structure could be fabricated by depositions of polysilicon [9], diamond [10], and silicon oxide [4] on isotropically etched cavities, or poly-crystalline diamond [11] on a micro-electro-discharge machining cavity. The gap between the shell and electrode is usually determined by the thickness of the sacrifice layer, which can be precisely controlled. Therefore, the gaps of structures fabricated by the CVD approach are usually small, ranging from 1.7 μm to 20 μm. However, the asymmetry of shells severely depends on the isotropic etching or micro-electro-discharge machining process. 

3D blowtorch molding [5,12], chemical foaming [13], and glassblowing [6,14] have been developed with favorable roughness and symmetry. These approaches are based on the blowing process under high temperature close to the film material softening point. Assembly with a locating stem is usually undertaken to align the independently fabricated shell and electrode, where a gap has been reported as 10.3–16.3 µm [5]. Various glassblowing processes with integrated electrodes have been developed with a wide range of capacitive gaps. A thermal coefficient mismatch between the blown shell and the cavity mold was used to create a gap of about 8 µm [7]. Deep glass dry etching was carried out to define the gap (>30 µm), followed by glassblowing and XeF_2_ releasing [15]. A kind of out-of-plane electrode architecture has been proposed, using photoresist as the sacrificial layers, whose thickness defined the gap (10.7 µm) [2,16]. Satellite spheres were fabricated when the shell was blown, serving as electro [6,14]. The minimum gap has been reduced to 5 µm. It should be noted that this kind of capacitor gap is composed of two crown faces with opposite orientations, resulting in a large equivalent gap distance. Annular electrodes were presented, to decrease the opposite area [17]. However, it was difficult to reduce the gap further by enlarging the sphere or annular electrodes, because they may vibrate as the electrostatic forces are applied. In that case, the excitation and detection of the shell resonator would be interfered with. Therefore, though favorable symmetry and roughness could be achieved by the blowing process, the gap is still relatively high. Overall, it is hard to realize a balance in both the symmetry and the gap.

**Figure 1:**
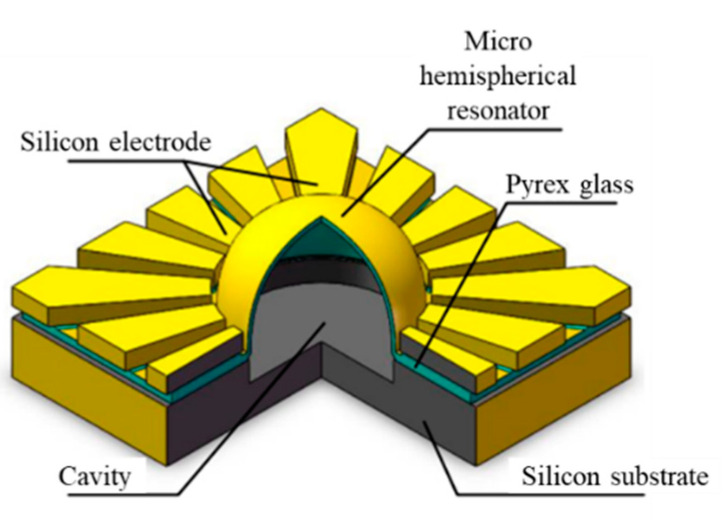
μHR structure illustration.

In this paper, a new μHR structure fabricated by a blowing process is proposed, which includes sandwich-shaped stacks and eave-shaped electrodes, as shown in Figure 1. The blowing process leaves the shell with favorable surface roughness and symmetry. In addition, sandwich-shaped stacks and eave-shaped electrodes make it feasible to realize uniform and small gaps. Therefore, the asymmetry, surface roughness, and gap could be kept at a low level, through the novel design in structure and fabrication process. Moreover, the electrical insulation of the shell and silicon-based electrodes could be guaranteed, owing to the eave-shaped structure. The various hemispherical shells are illustrated in Table 1.

## 2. Design

**Figure 2:**
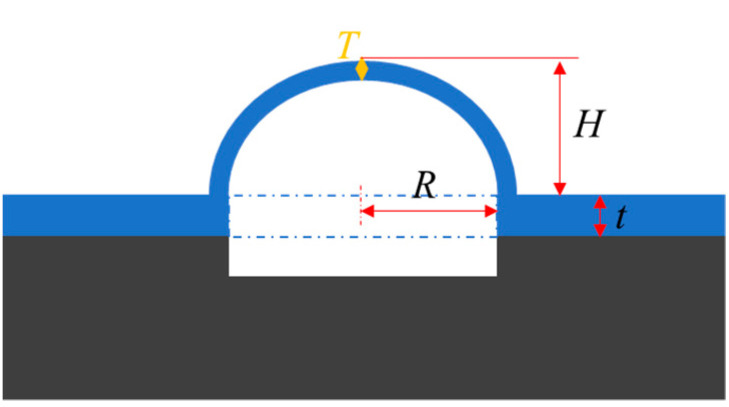
Dimensional sketch of hemispherical shell.

To investigate the influence of dimension parameter on the performance, simulation models are established by COMSOL Multiphysics® 5.6 (COMSOL, Inc., Burlington, MA, USA). The dimensional sketch of the hemispherical shell is illustrated in Figure 2. The bottom cross-section is circular. 

**Figure 3:**
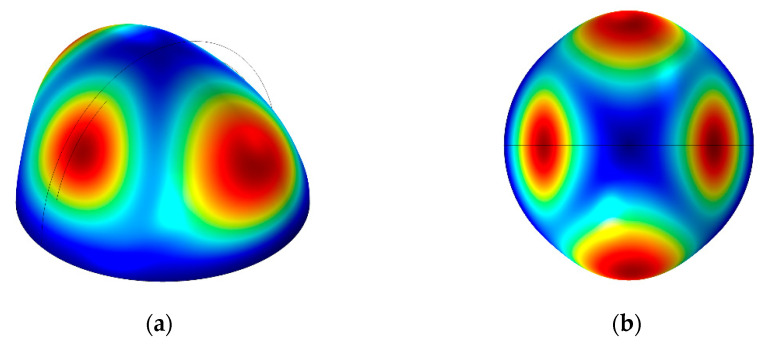
1st m = 2 mode of HRG (**a**) inclined view (**b**) overview.

Finite element modal analysis is undertaken to find the vibrating-mode shapes of shells and the resonant frequency. When the shell works in 1st m = 2 wineglass mode, there are four uniformly distributed polar zones, as shown in Figure 3. The thickness of the shell is set as 50 µm. The resonant frequency of the hemispherical shell with different radius could be simulated, as shown in Table 2.

**Figure 4:**
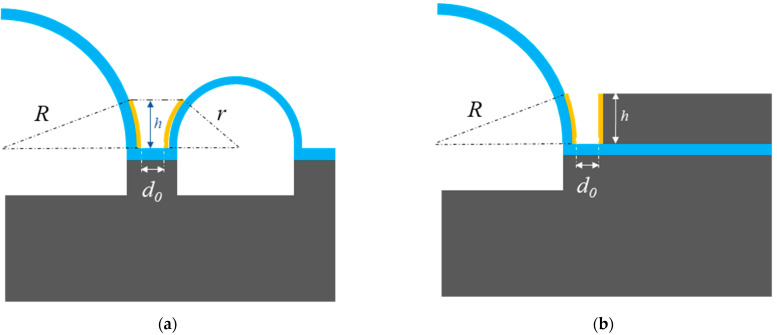
Comparison of μHRs with annular electrodes and silicon-based electrodes (**a**) annular electrodes; (**b**) silicon-based electrodes.

The structure consists of a 3D micro-hemispherical shell and 16 surrounding discrete electrodes, as shown in Figure 4. This part will focus on the silicon-based electrodes. Here, annular electrodes [17] and silicon-based electrodes are compared.

The capacitor could be simplified as two parallel plate electrodes which are separated by a vacuum. 

For the annular electrode, the capacitor value $C_{a}$ could be expressed as:

(1) $$C_{a}=\int_{0}^{h}\frac{\varepsilon_{0}w}{d_{0}+R-\sqrt{R^{2}-y^{2}}+r-\sqrt{r^{2}-y^{2}}}dy$$

where *h* is the effective height of the electrodes, $\varepsilon_{0}$ is the permittivity of the medium in vacuum, *w* is the effective width of the electrodes, and $d_{0}$ is the gap between the bottom of electrodes, *R* is the radius of hemispherical shell, *r* is the radius of annular electrode, *y* represents the variable symbol along the coordinate axes of electrode height. 

Correspondingly, the capacitor value for the silicon-based electrode $C_{s}$ could be expressed as:

(2) $$C_{s}=\int_{0}^{h}\frac{\varepsilon_{0}w}{d_{0}+R-\sqrt{R^{2}-y^{2}}}dy$$

It should be noted that $C_{s}$ would be larger than $C_{a}$, assuming the dimensional parameters *h*, *w*, and *R* are the same. The radius of annular electrode *r* is set to be a relatively small value, to guarantee that the resonant frequency of the annular electrode is much bigger than that of the hemispherical shell [17]. 

**Figure 5:**
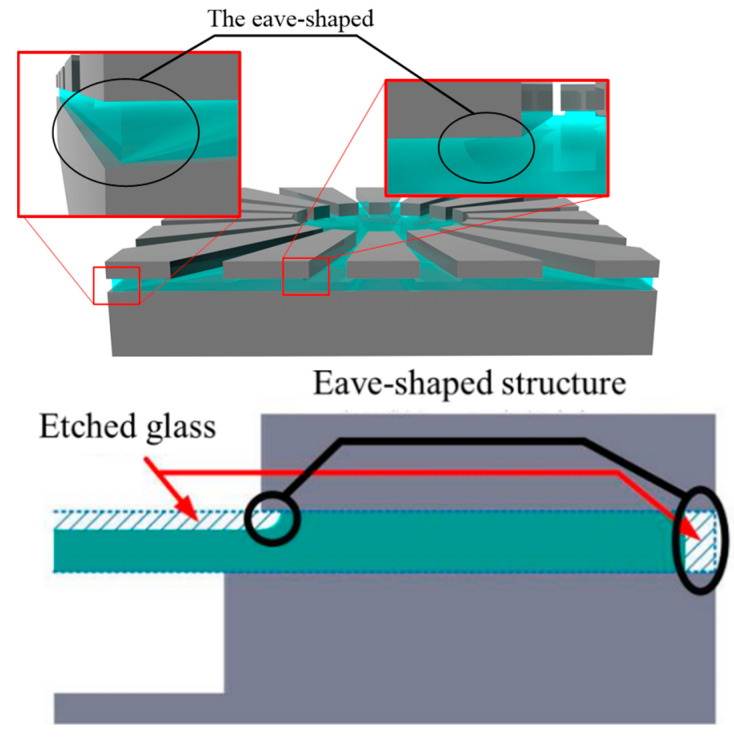
Illustration of the eave-shaped structure.

Next is the fabrication process of the eave-shaped electrode. The top silicon layer of the sandwich-shaped stacks (anodically bonded silicon–glass–silicon) are etched by deep reactive ion etching (DRIE) technique to form the silicon electrode. After that, the wafer is soaked in the HF (40%) to form an eave-shaped structure owing to isotropic etching of glass, as shown in Figure 5. There are two reasons to design eave-shaped silicon electrodes. One is that silicon can be fabricated in an anisotropic way. Therefore, the shape and dimension of the electrode can be precisely determined, which is favorable for reducing the gap between the shell and eave-shaped electrodes. The other is that the eave-shaped structure could be easily formed because silicon could be bonded with glass, and the etch solution of glass is not reactive with silicon. Therefore, self-insulting of electrodes could be realized. 

## 3. Fabrication Process

**Figure 6:**
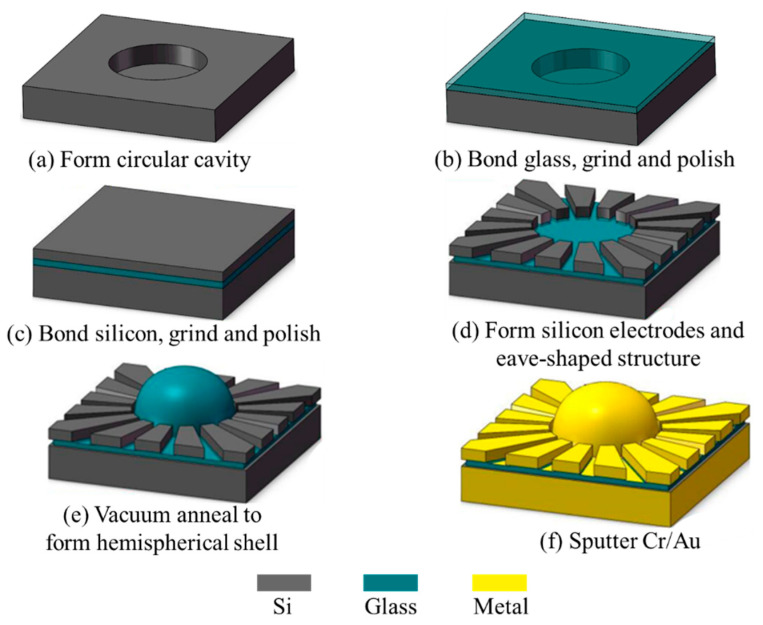
Fabrication process sketch.

The forming process of the hemispherical shell resonator uses the different pressure between the inside and outside of the hermetic cavity and the surface tension forces from the softened glass. The fabrication of the hemispherical shell resonator with integrated silicon-based electrodes is illustrated as Figure 6: (a) A 500 μm-thick silicon wafer is etched using the DRIE technique with 7 μm-thick AZ4620 photoresistor (Microchemicals GmbH, D-89079, Ulm, Germany) as a pattern mask, to form a 100 μm-deep circular cavity. (b) After removing the photoresistor and cleaning the silicon wafer, anodic bonding of a new 525 μm-thick Pyrex 7740 glass wafer (Corning Inc., New York, NY, USA) and the silicon wafer is performed, where the circular cavities are encapsulated with atmospheric air. Then, the glass wafer is ground and polished using a chemical mechanical polishing (CMP) technique until the thickness reaches 100 μm. Lithography is performed on the backside silicon surface and a 10 μm-deep cross channel is formed by DRIE, to provide an alignment label for subsequent processes. (c) Another new 400 μm-thick silicon wafer is anodically bonded with the glass surface of the former stacked wafer. The top silicon layer is ground and polished by CMP until the thickness reaches 100 μm. (d) a 7 μm-thick AZ4620 photoresistor (Microchemicals GmbH, D-89079, Ulm, Germany) is spun on the top silicon layer, and lithography is carried out by aligning the backside cross channel. Then the top silicon layer is etched by DRIE, until the Pyrex glass layer is exposed to form silicon-based electrodes. After that, the photoresistor is removed. The wafer is immersed in the HF (40%) for 8 min to etch the glass beneath the silicon-based electrodes and form the eave-shaped structure; (e) then, vacuum annealing is conducted, with a vacuum degree of 20 m Torr, furnace temperature of 770 °C and a remaining time of 2 min, and then the furnace temperature is slowly decreased to 200 °C in 5 min. The glass film is blown and solidified to form a hemispherical shell. (f) 50 nm/100 nm-thick Cr/Au is deposited on the whole wafer surface using the magnetron sputtering technique, thus the silicon-based electrodes and shell metals are automatically separated owing to the non-conformal metal deposition on the eave-shaped structure. The challenge of the fabrication technology is to control the parameter of blowing process to realize a uniform shell and small gap. The annealing temperature and vacuum degree of the blowing process should be delicately investigated.

## 4. Fabrication Result

The key process is the formation of the surrounding eave-shaped electrodes. The lateral etching volume of the Pyrex glass beneath the eave-shaped electrodes should be well manipulated. If the eave is too shallow, the metal may be conformally deposited on the glass sidewall, resulting in short-circuiting between the electrodes. If the eave is too deep, the contact area between the eave-shaped electrodes and the glass would be dramatically reduced due to double-side etching. This is unfavorable when conducting glassblowing experiments.

**Figure 7:**
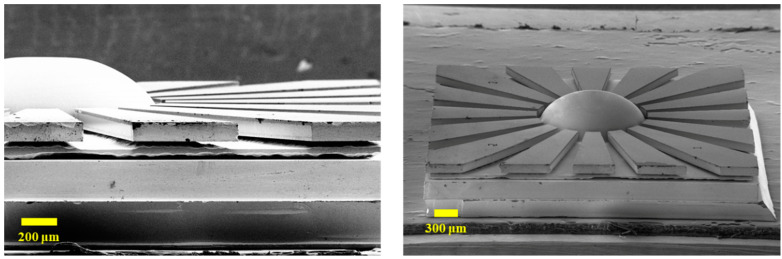
SEM pictures of the eave-shaped structure.

The etching rate of Pyrex glass in HF solution (40%) is measured as 3.7 μm/min. In addition, the appropriate lateral etching width is designed to be about 30 μm, which could be realized after immersing the wafer in HF solution for 8 minutes. The SEM pictures of the eave-shaped structure are shown in Figure 7.

**Figure 8:**
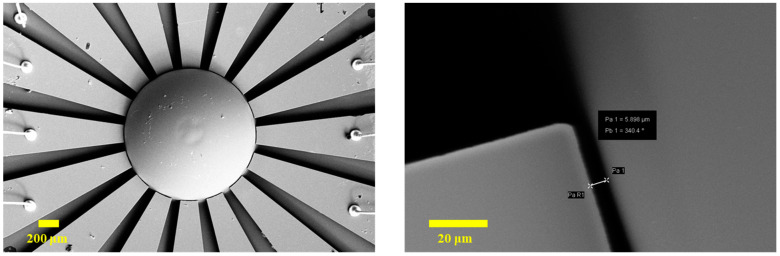
SEM pictures of μHR.

The big challenge in the process is to blow up the glass to form a hemispheric shell with an extremely small gap in the eave-shaped electrode. The shell shape is determined by the internal–external pressure difference and the annealing temperature. Vapor pressure force, interfacial force, and gravity force contribute to the blowing process. The vacuum of the annealing furnace is kept at 20 m Torr. The temperature is raised up to 770 °C, close to the softening temperature of Pyrex 7740 glass, and maintained for 2 min. Then, the temperature slowly falls to 200 °C over 5 min. The fabricated μHR is illustrated in Figure 8, with a gap down to 5.9 μm from shell to electrode. 

**Figure 9:**
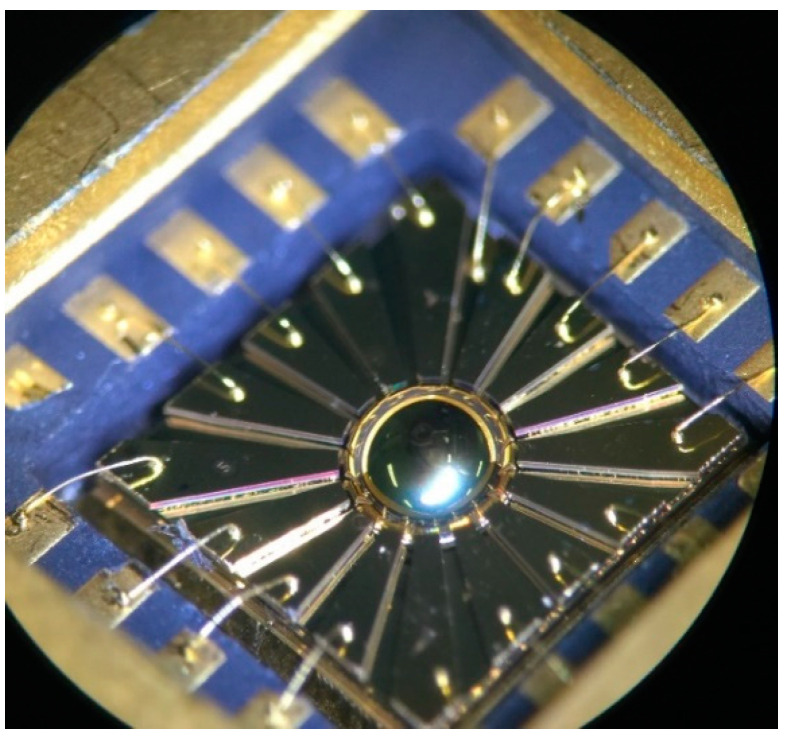
Physical photo of μHR after wire-bonding.

A physical photo of the μHR after wire-bonding is shown in Figure 9. The micro-hemispherical shell and 16 surrounding discrete electrodes can be clearly observed. After wire-bonding, the electrodes are connected to the ceramic package for signal processing, which are verified to have insulated each other.

**Figure 10:**
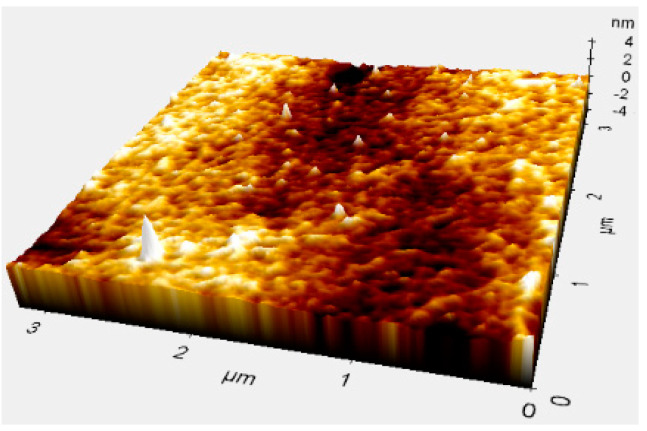
Atomic force microscopy (AFM) graph of surface.

The surface roughness of the hemispherical shell is crucial for the vibration quality factor. An atomic force microscope (AFM) measurement was performed to characterize the surface roughness after glassblowing and metal deposition, as shown in Figure 10. The roughness value was 0.26 nm ± 0.06 nm, which was comparable to the previously reported values ranging from 0.22 nm to 2 nm, achieved via the glassblowing process, and this was prior to those of other molding processes, as mentioned in Table 1. 

## 5. Nondestructive Estimation of Shell Thickness

The blowing process is usually performed to fabricate a glass hemispherical shell. The thickness of the spherical shell is critical to the resonant frequency and sensitivity of the μHR. However, to measure the thickness, the glass shell would have to be broken along its vertical section. Herein, it is necessary to develop an estimating method in a nondestructive manner. We assume that the volume of the blowing glass is constant. That means the plane volume is equal to the spherical shell one, which could be considered to be the volume difference of two spherical caps.

(3) $$\pi R^{2}t=\frac{\pi }{6}H[H^{2}+3{\left(R+T\right)}^{2}]-\frac{\pi }{6}(H-T)[{\left(H-T\right)}^{2}+3R^{2}]$$

The parameters are labeled in Figure 2. This could be simplified as the cubic equation:

(4) $$T^{3}+(3H^{2}+6HR+3R^{2})T-6R^{2}t=0$$

**Figure 11:**
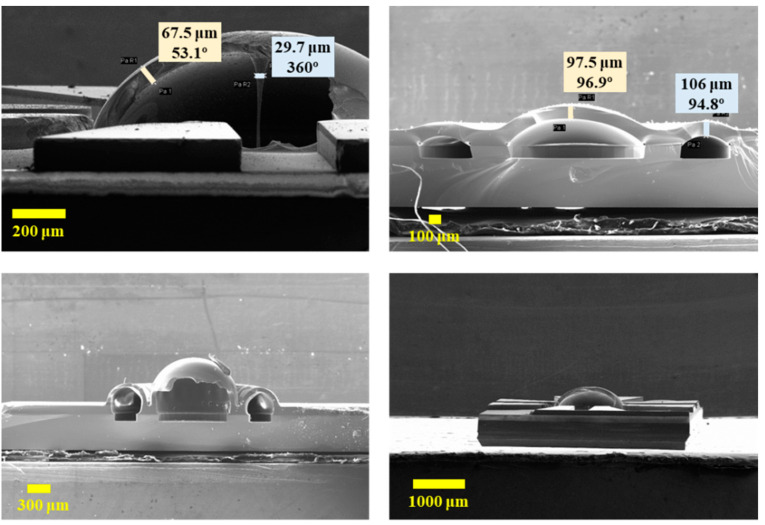
SEMs of broken hemispherical shells.

The positive solution of this cubic equation can be taken as the estimated thickness value, noted as *T_f_*. Typical shell thickness *T_m_* can be measured via SEM, as shown in Figure 11, where the hemispherical shells are destroyed. 

The parameters are compared in Table 3. The cavity radius “*R*” can be extracted from the layout design, which can also be confirmed by optical microscopy. The height of shell “*H*” and the initial thickness of glass “*t*” can be measured by optical microscopy via the focusing method. The deviations between estimated thickness value (*T_f_*) and measured value (*T_m_*) are within the acceptable range. These results demonstrate that the estimation method can realize nondestructive testing of hemispheric shell thickness with deviation below 5%.

## 6. Conclusions

This μHR structure based on a hemispherical shell and surrounding eave-shaped electrodes is presented in this paper. It possesses low asymmetry and surface roughness, and a uniform and small gap, compared to the previously reported μHR structure fabricated by molding deposition, precise machining, and glassblowing. The fabrication process is presented, including the formation of eave-shaped silicon-based electrodes and a hemispheric shell. The shape and dimensions of the electrode can be precisely determined, making the gap between the shell and eave-shaped electrodes controllable. The eave-shaped structure is formed to obtain self-insulting of electrodes, where the appropriate lateral etching width is about 30 μm. The hemispheric shell is blown, with a small capacitor gap down to 5.9 μm, which is competitive among the reported μHR s via a blowing process. The asymmetry and surface roughness after glassblowing were measured as 0.04% and 0.26 nm, respectively, which are comparable to the reported values via the glassblowing process. Finally, an estimation method of shell thickness in a nondestructive manner is developed, with deviation below 5%. These results preliminarily indicate that this structure with a hemispherical shell and surrounding eave-shaped electrodes is promising in μHR application. Further investigation of the performance characterization of μHRs should be carried out.

## Figures and Tables

**Table 1:** Various hemispherical shells.

Source	Shell Material	Fabrication Method of Shell	Electrode Shape	Alignment of Shell and Electrode	Surface Roughness (nm)	Asymmetry	Typical Hemisphere Radius (μm)	Capacitive Gap (μm)
X. Zhuang et al. [9]	Polysilicon	isotropic etching, CVD, sacrificing	Synclastic hemisphere	Integration	--	0.55%	650	1.7
P. Pai et al. [4]	Silicon oxide	isotropic etching, CVD, sacrificing	Plane on silicon	Integration	<5	2.2%	250	10
D. Horsley et al. [11]	Diamond	µEDM, CVD, sacrificing	No electrode	--	4	2.3%	500 ± 6	--
K. Najaf et al. [5,12]	Fused silica	fuel-oxygen blowtorch	On vertical silicon sidewall	Assemble with locating stem	--	/	2500	10.3–16.3
B. Sarac et al. [18]	Metallic Glass	Blow Molding	No electrode	--	<2	7.8%	500	--
M. Kanik et al. [7]	Metallic Glass	Blow Molding	Synclastic hemisphere	Integration	0.3	0.66%	1500	8
J. Xie et al. [13]	BF-33 glass	Chemical Foaming Method	No electrode	--	--	0.12%	544	--
A. Shkel et.al. [15]	Glass	Glassblowing	On vertical glass sidewall	Integration	--	/	2200	>30
A. Shkel et.al. [2,16]	Fused silica	Glassblowing	Plane on silicon	Integration	0.23	/	3500	10.7
A. Shkel et al. [6,14]	Pyrex glass	Glassblowing	Satellite sphere fabricated by glassblowing	Integration	0.85	0.05%	500	5 (minimum)
R. Wang et al. [17]	Pyrex glass	Glassblowing	Annular electrode	Integration	0.22	/	500	73
This work	Pyrex glass	Bonding, glassblowing	On vertical silicon sidewall	Integration	0.26	0.04%	275	5.9

“--” represents “Not mentioned in the paper”. “/” represents “could Not obtained from the paper”.

**Table 2:** Resonance frequency of shell with different radius.

Bottom Radius of Shell (μm)	500	525	550	575	600
1st m = 2 mode resonance frequency (kHz)	1.58 × 10^3^	1.49 × 10^3^	1.40 × 10^3^	1.33 × 10^3^	1.26 × 10^3^

**Table 3:** Parameter list and comparison.

*R*	*H*	*t*	*T_m_*	*T_f_*	Deviation
550	455	109	67.5	65.2	3.4%
550	321	120	97.5	95.3	2.2%
550	648	120	53.1	50.6	4.7%
600	464	120	76.6	76.2	0.5%

## Data Availability

The data presented in this study are available on request from the corresponding author. The data are not publicly available due to technique privacy.
